# Non-small cell lung cancer: the new T1 categories

**DOI:** 10.12688/f1000research.10600.1

**Published:** 2017-02-22

**Authors:** Paul E. Van Schil

**Affiliations:** 1 Department of Thoracic and Vascular Surgery, Antwerp University Hospital, Antwerp, Belgium

**Keywords:** Lung cancer, TNM staging system, TNM classification, T1 lesions, Cancer staging, TNM Classification of Malignant Tumours, Classification of Malignant Tumours

## Abstract

Recently, major changes have occurred in the staging, diagnosis, and treatment of early stage lung cancer. By screening high-risk populations, we are now able to detect lung cancers at an early stage, but the false-positive rate is high. A new pathological classification was published in 2011 and fully incorporated in the 2015 World Health Organisation (WHO) Classification of Tumours of the Lung, Pleura, Thymus, and Heart. The new eighth edition of the tumour–node–metastasis (TNM) staging system has been fully published and will be in use from January 2017. T1 lesions are subdivided into T1a, T1b, and T1c lesions corresponding to lung cancers up to 10 mm, between 11 and 20 mm, and between 21 and 30 mm, respectively. To determine the size, only the solid part on computed tomographic scanning of the chest and the invasive part on pathological examination will be considered. Prognosis is significantly better for the smallest lesions. For some specific subgroups, sublobar resection may be oncologically valid and yield good long-term outcome, but the results of recently performed randomised trials are awaited.

## Introduction

This century is quite exciting for thoracic oncologists and thoracic surgeons as they are confronted with new challenges. Screening high-risk populations has become a hot topic to detect lung cancer at an early stage. However, how to manage screen-detected nodules remains a matter of intense debate, as quite a lot of false-positive results are encountered on initial screening studies ^1^. Especially from Japanese studies, it became clear that for small, very early lung cancers, particularly of the adenocarcinoma subtype, a limited resection may provide good long-term results ^2, 3^. This is reflected in the new edition of the tumour–node–metastasis (TNM) classification, with further subdivisions of the T descriptor ^4^. In 2015, a new World Health Organisation (WHO) Classification of Tumours of the Lung, Pleura, Thymus, and Heart was introduced with special emphasis on new subcategories of adenocarcinomas ^5, 6^. In this review, I will describe the new T1 categories and highlight the different subtypes of adenocarcinomas with a focus on early stage lesions. Also, I will address the current role of limited resection for early stage lung cancer.

## T1 descriptor in the eighth TNM classification

The eighth edition of the TNM classification is based upon the large database of the International Association for the Study of Lung Cancer (IASLC) comprising prospective and retrospective data from all continents except Africa ^7, 8^. Most data originate from Asia and Europe. Officially, this new TNM classification has to be applied from January 2017, but for logistic reasons in North America this will only be done from January 2018.

When analysing this new version, regarding clinical as well as pathological (postoperative on resected specimens) staging, it became clear that tumour size is an important prognostic factor, allowing further subdivisions of the T category ^4^. In contrast to the previous edition where T1a comprised tumours up to 20 mm, the current T1 and T2 descriptors are composed of subcategories with 1 cm intervals ( Table 1). This provides a more logical order for clinicians. It should also be noted that to determine the current T size, only the invasive part of the tumour is considered for the clinical as well as pathological description ^9^. This is especially important for subsolid lesions where the solid part measured on lung window settings of computed tomographic (CT) scanning corresponds in most cases to the invasive part measured by pathological examination.

**Table 1.** T1 and T2 categories according to size in the seventh and eighth edition of the tumour–node–metastasis (TNM) classification ^4, 29^.

T category	Seventh TNM edition	Eighth TNM edition
T1a	≤20 mm	≤10 mm
T1b	21–30 mm	11–20 mm
T1c	-	21–30 mm
T2a	30–50 mm	30–40 mm
T2b	51–70 mm	41–50 mm

The subdivision of the T1 category has prognostic implications, as 5-year survival rates for clinical staging were 92%, 83%, and 76% for T1a, T1b, and T1c cancers, respectively ^4^. For pathological staging, 5-year survival rates were 91%, 86%, and 81% for T1a, T1b, and T1c lesions, respectively.

## WHO 2015 classification of lung tumours

In 2011, a common task force including a broad range of thoracic specialists from the IASLC, American Thoracic Society (ATS), and European Respiratory Society (ERS) proposed a new adenocarcinoma classification with incorporation of several new subcategories to provide a clinically useful system agreed upon not only by pathologists but also by thoracic radiologists, pulmonary physicians, medical and radiation oncologists, and thoracic surgeons ^10^. In this way, relevant diagnostic and therapeutic algorithms can be created related to specific pathological entities. A distinction was made between small biopsy specimens and resected specimens, the latter allowing more extensive immunohistochemical testing and mutation analysis. Specific handling of these specimens has been described in detail ^11^.

Regarding smaller lesions of ≤30 mm, new subcategories include adenocarcinoma *in situ* (AIS) and minimally invasive adenocarcinoma (MIA). AIS is defined as a non-invasive lesion that has a maximum diameter of 30 mm and a purely lepidic pattern. This corresponds mostly to a ground-glass nodule (GGN) on CT scan. When the lesion is completely resected, disease-free survival is 100%. MIA also has a predominantly lepidic pattern and ≤5 mm invasion in greatest dimension in any one focus without signs of necrosis. Invasive adenocarcinomas are subdivided into lepidic predominant, acinar, papillary, micropapillary, and solid variants. Mixed tumours should be described semi-quantitatively in 5% increments choosing a single predominant histologic pattern. The term “bronchioloalveolar carcinoma” (BAC) is not used anymore, as it gave rise to much confusion with several different definitions utilised throughout the world. It should be noted that solid lesions described as such on chest CT scan do not necessarily correspond to solid adenocarcinomas, as the latter are a particular subdivision of invasive adenocarcinomas.

From several phase II studies, it became clear that AIS and MIA have an excellent prognosis when completely resected with no vascular or lymph node involvement ^12^. In contrast, solid and micropapillary variants have a worse prognosis with a higher incidence of locoregional recurrences, and this should be taken into account when deciding on the extent of resection for these specific subtypes.

The natural history of early lesions discovered on CT scanning has to be further elucidated. In a prospective study of 1,229 subsolid nodules comprising 100% GGN and part-solid lesions, a mean follow-up period of 4.3 years was reached ^13^. Regarding the pure GGNs, only 1.2% developed into heterogeneous variants and 5.4% into part-solid nodules. Invasive adenocarcinomas were detected only in the subgroup of part-solid nodules, corresponding to 1% of the whole series.

A recently recognised pathological entity is the so-called “spread through air spaces” (STAS), consisting of separate malignant cell clusters around the primary lesion but not in direct contact with the main tumour ^14^. This specific variant has a higher risk of local recurrence in case of limited resection, compromising long-term survival.

## Limited resection of early stage lung cancer

With the positive results of the National Lung Screening Trial (NLST) showing a clear advantage of CT screening in high-risk populations compared to standard radiographs, the question arose of whether lobectomy is indicated for all tumours or whether very early stage lesions can be treated by so-called limited or sublobar resections ^15, 16^. These comprise wide wedge excision with the use of stapling devices and purely anatomical segmentectomies, which are technically more difficult to perform, especially by minimally invasive techniques. Also, the need for systematic nodal dissection as defined by a working group of the IASLC has been questioned for these early stage lesions ^17^. Many phase II studies, most of them originating from Japan, indeed showed that GGNs, pathologically corresponding to AIS and MIA in most cases, can be treated with a limited resection, yielding 5-year disease-free survival rates exceeding 95%. Vascular invasion and lymph node invasion are very rarely encountered, so extensive lymph node dissection is probably not routinely necessary ^3, 18^. Results from meta-analyses are somewhat conflicting but, generally, good long-term results are described for tumours ≤20 mm treated by segmentectomy when no lymph node invasion is present. However, for small, early stage lung cancer, no high-level grade A evidence is currently available. The only randomised trial that has been fully published dates back to 1995 and was performed by the Lung Cancer Study Group (LCSG) with updated and corrected results published one year later ^19, 20^. At that time, no high-resolution CT scanning or positron emission tomographic (PET) scanning was available. Lesions up to 3 cm were intraoperatively randomised between classical lobectomy and sublobar resection. Half of the patients had a contraindication to randomisation because of the size of the tumour or lymph node involvement at the hilar or mediastinal region. Both segmentectomy and wedge resection were allowed. Although only marginally significant, disease-free survival was better for the lobectomy group, which worldwide became the standard intervention for lung cancer resection, even for smaller lesions ^19^. Two new randomised trials address the same question but they include only tumours up to 20 mm. The Japanese study JCOG0802/WJOG4607L trial has recently been closed for inclusion, as the target of 1,100 patients was reached, and long-term results are awaited ^21^. The North American CALGB 140503 phase III trial is still accruing patients ^22^. So, it will still take several years before the long-term results of these trials become available.

Subcentimeter lung cancers, currently T1a disease, represent a specific subgroup, as they comprise the smallest lesions ^23^. For this reason, they have become a specific focus of interest. In a series of 291 patients who underwent resection of a subcentimeter lung cancer, a subdivision into four categories was made ranging from 100% ground-glass opacities (GGO, non-solid lesions) to 0% GGO (purely solid lesions) ^24^. As can be anticipated, adenocarcinoma was the most common pathological diagnosis. Lymph node metastases were present only in solid lesions. In the latter subcategory, the highest incidence of recurrent disease and lowest overall and disease-free survival were noted. The authors concluded that lobectomy should still be performed for purely solid lesions but sublobar resection may be considered for the other categories.

For thoracic surgeons, another important issue is the accuracy of intraoperative frozen section analysis to determine the extent of resection. Recent studies point out that a concordance rate of more than 80% can be reached between the frozen section and definitive pathological report ^25, 26^. However, AIS and MIA are more difficult to diagnose on frozen section, and accuracy is lower for lesions below 10 mm, which in fact represent the main category to be considered for sublobar resection. This implies that a second intervention to perform a completion lobectomy may be indicated in patients with unfavourable histology who initially underwent a sublobar resection for a presumably low-malignant lesion.

## Perspective

Diagnosis and treatment of early stage lung cancer are constantly evolving as new data steadily become available. Screening trials have shed new light on screen-detected nodules. The new TNM classification provides specific subcategories with a different prognosis. Pathological classification includes new subdivisions delineating early stage lesions as in breast cancer.

Specific guidelines that can be generally applied become necessary to create diagnostic and therapeutic algorithms adapted to the smallest lung cancers. Primarily intended for thoracic surgeons, the Society of Thoracic Surgeons (STS) installed a task force to optimize therapy of screen-detected lung lesions and minimize morbidity of false-positive diagnoses ^1^. Recently, the European Society of Thoracic Surgeons (ESTS) made recommendations for the implementation of CT screening in Europe, taking into account not only the training of thoracic surgeons and their clinical profile but also the use of minimally invasive thoracic surgery, which is more widely applied at the current time ^27^. Also, the Fleischner Society updated its guidelines for the diagnosis and management of small pulmonary nodules detected on chest CT scans ^28^.

In this way, this new area in thoracic oncology and surgery will continue to remain a hot topic at major conferences worldwide but will be more precisely defined in the years to come, providing guidelines that are universally accepted and applied by international surgical and oncological societies.

## Abbreviations

AIS, adenocarcinoma *in situ*; CT, computed tomography; GGN, ground-glass nodule; GGO, ground-glass opacity; IASLC, International Association for the Study of Lung Cancer; MIA, minimally invasive adenocarcinoma; TNM, tumour–node–metastasis; WHO, World Health Organisation.
